# Expression of Concern: De Bari et al. Could 18-FDG PET-CT Radiomic Features Predict the Locoregional Progression-Free Survival in Inoperable or Unresectable Oesophageal Cancer? *Cancers* 2022, *14*, 4043

**DOI:** 10.3390/cancers17091400

**Published:** 2025-04-23

**Authors:** Cancers Editorial Office

**Affiliations:** MDPI, St. Alban-Anlage 66, 4052 Basel, Switzerland; cancers@mdpi.com

The *Cancers* Editorial Office wishes to alert readers of concerns regarding the ethical approval of this study [1]. Following publication, concerns were raised regarding inconsistencies in the ethical review information reported in this article [1]. Although the name of the institution responsible for ethical review was provided, the date of approval remains unclear. The authors and affiliated institution have been contacted and were requested to clarify the ethical approval information for this article, but remain unresponsive. This notice is issued to ensure transparency and to maintain the integrity of the scholarly record. No further editorial action is planned at this time. The Journal may reconsider this decision should additional relevant information become available.
