# Supplementary material for: Could 18-FDG PET-CT Radiomic Features Predict the Locoregional Progression-Free Survival in Inoperable or Unresectable Oesophageal Cancer?
Source: Cancers (Basel). 2022 Aug 22;14(16):4043. doi: 10.3390/cancers14164043 (PMC9406583; doi:10.3390/cancers14164043)
Supplement: Supplementary file 1 [file cancers-14-04043-s001.zip › Supplementary material Table 1.pdf]

Table 1. List of the 55 variables that were used in the heat map definition. Please, refer to [23] for a precise definition of each variable.
L_minor
F_stat_entropy
L_least
F_rlm_merged_glnu
F_rlm_2_5D_glnu
F_szm_2_5D_glnu_norm
F_szm_2_5D_gl_var
F_rlm_2_5D_rlnu
F_szm_2_5D_glnu
F_szm_2_5D_zs_var
F_cm_2_5D_clust_shade
F_stat_median
F_szm_zsnu
F_stat_energy
F_rlm_rl_entr
F_rlm_2_5D_rl_entr
F_cm_2_5D_info_corr_2
F_cm_2_5Dmerged_info_corr_2
F_cm_2_5D_diff_var
F_rlm_2_5D_sre
F_zsm_z_perc
F_szm_sze
F_rlm_merged_sre
F_cm_inv_diff_mom_norm
F_cm_2_5D_inv_diff_mom_norm
F_cm_merged_inv_diff_norm
F_cm_2_5D_inv_diff_norm
F_stat_Nic_entropy
F_stat_min

F_szm_lzhge
F_szm_zs_var
F_rlm_2_5D_rl_var
F_szm_lzlge
F_szm_2_5D_z_entr
F_morph_com
L_major
F_cm_2_5D_joint_max
F_cm_info_corr_1
F_rlm_r_perc
F_stat_kurt
F_szm_zsnu_norm
F_cm_energy
F_szm_szlge
F_rlm_2_5D_srlge
F_rlm_merged_lrlge
F_rlm_2_5D_lrlrlm_25D_merged_df
F_cm_2_5D_info_corr_1
F_stat_uniformity
F_rlm_glnu_norm
F_morph_av
F_cm_2_5D_inv_var
F_cm_inv_var
F_morph_sphericity
F_morph_pca_elongation
F_morph_pca_flatness
