# Supplementary figures and images for: Could 18-FDG PET-CT Radiomic Features Predict the Locoregional Progression-Free Survival in Inoperable or Unresectable Oesophageal Cancer?
Source: Cancers (Basel). 2022 Aug 22;14(16):4043. doi: 10.3390/cancers14164043 (PMC9406583; doi:10.3390/cancers14164043)

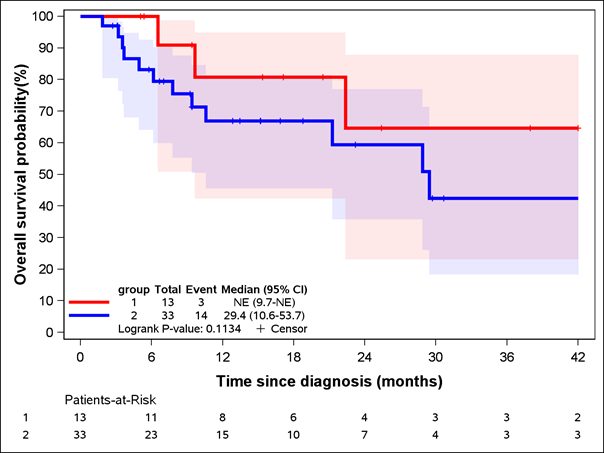

Supplement: Supplementary file 1 [file cancers-14-04043-s001.zip › Suppl Fig 1 OS by groups.tif]

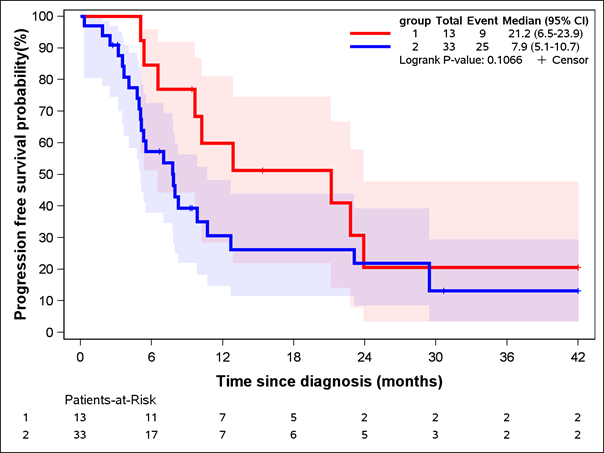

Supplement: Supplementary file 1 [file cancers-14-04043-s001.zip › Suppl Fig 2 PFS by groups.tif]

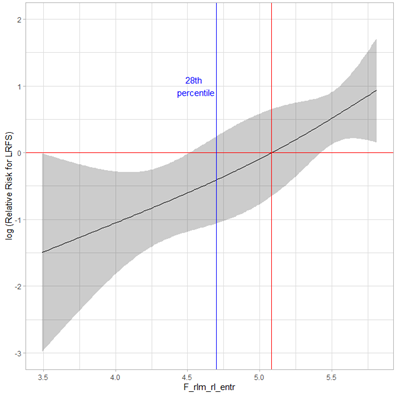

Supplement: Supplementary file 1 [file cancers-14-04043-s001.zip › Suppl Fig 3 threshold of F_rlm_rl_entr.tif]

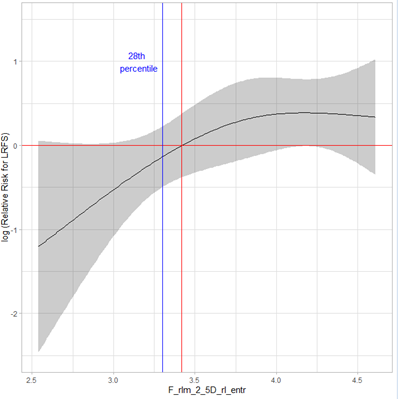

Supplement: Supplementary file 1 [file cancers-14-04043-s001.zip › Suppl Fig 4 threshold of F_rlm_2_5D_rl_entr.tif]

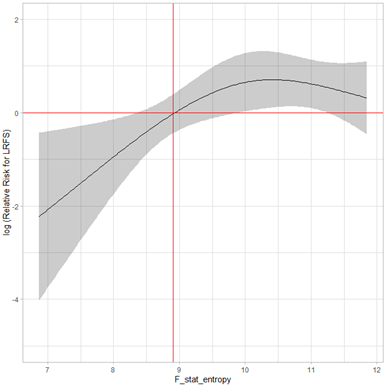

Supplement: Supplementary file 1 [file cancers-14-04043-s001.zip › Suppl Fig 5 threshold of F_stat_entr.tif]

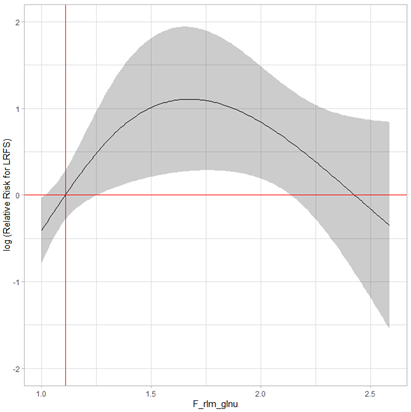

Supplement: Supplementary file 1 [file cancers-14-04043-s001.zip › Suppl Fig 6 threshold of F_rlm_glnu.tif]

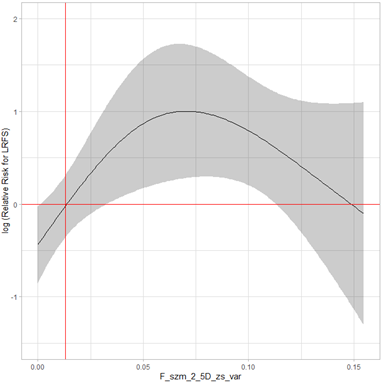

Supplement: Supplementary file 1 [file cancers-14-04043-s001.zip › Suppl Fig 7 threshold of F_szm_2_5D_zs_var.tif]
